# Hospital environment and patient recovery – a review

**DOI:** 10.1192/j.eurpsy.2022.1946

**Published:** 2022-09-01

**Authors:** A. Lourenço, M. Ribeiro, M. Lemos

**Affiliations:** Centro Hospitalar Lisboa Norte, Psychiatry, Lisboa, Portugal

**Keywords:** hospital environment influence, patients’ recovery, patients healing

## Abstract

**Introduction:**

Since the second half of the 20th century, studies have been carried out that prove the benefits of the hospital environment in the improvement and recovery of patients. In this way, it would be important to understand what has already been done within the reality of Psychiatry Department.

**Objectives:**

To review the literature about the documentation of hospital environment influence in patients’ recovery.

**Methods:**

We performed a MEDLINE search using the key words: hospital environment influence and patients’ recovery or patients healing. We only included studies with full text published in English.

**Results:**

In the selected articles, we only found studies developed in the Surgery department; in one of them, the authors tested the presence of plants in the patients’ rooms and assessed lower blood pressure values, less pain and less anxiety than the control group; in another, they tested the presence of music and landscape, although there was no difference in terms of pain assessment, there was an improvement in the assessment of the postoperative experience. A review pointed some other aspects that patients linked with their recovery, such as: audio and visual environment; specifically, in anxiety, pain and stress. On the other hand, other studies address the influence of the hospital environment on the satisfaction of health care providers.

**Conclusions:**

Although hospital environment has already demonstrated an impact on the patient recovery, none (in our review) was developed directly in a Psychiatric Department; further studies are needed to understand the impact on this kind of service.

**Disclosure:**

No significant relationships.

